# 

**Published:** 1903-10-03

**Authors:** 


					^ "**" stau ' purity. .av,.
^aDBURY s rt- t-JIfc"* ?. .Y'S
cocoa \ Jni - 1 V- -.a
" A PERFECT FOOD." <*-* ?WO DRUG . ALKALIES Ugxn
No. 888.. Vol. XXXV. Saturday,
Oct. 3rd, 1903.
rXn4Tcrms'] PRUJE threepence.

				

